# Sex difference in the expression of PD-1 of non-small cell lung cancer

**DOI:** 10.3389/fimmu.2022.1026214

**Published:** 2022-10-20

**Authors:** Yong Gu, Ying Y. Tang, Jian X. Wan, Jian Y. Zou, Chuan G. Lu, Hao S. Zhu, Si Y. Sheng, Yan F. Wang, Hai Ch. Liu, Jia Yang, Hai Hong

**Affiliations:** ^1^ Department of Thoracic Surgery, The First Affiliated Hospital of Sun Yat-Sen University, Guangzhou, China; ^2^ Key Laboratory of Tropical Disease Control of Sun Yat-Sen University, Ministry of Education, The Institute of Immunology of Zhong Shan Medical School, Sun Yat-Sen University, Guangzhou, China; ^3^ Department of Cardiothoracic Surgery, Sanya Central Hospital, Sanya, China; ^4^ Department of Basic Medicine, Xiangnan University, Chenzhou, China

**Keywords:** NSCLC, sex difference, immunotherapy, soluble PD-1, membrane PD-1 expression

## Abstract

Evidence increasingly indicated that lung cancer incidence in female individuals continue to rise, and women have a higher risk to develop adenocarcinoma than men. Male and female individuals differ in their innate and adaptive immune responses, and there are sex differences in response to the PD-1/PD-L1-dependent blocking immunotherapy. Whether the differential expression of PD-1 between genders affect the response to blocking treatment is currently unknown. In this study, we examined sex differences in serum sPD-1, mPD-1 expression on T cells, and sex hormone levels in non-small cell lung cancer (NSCLC) patients. Our results revealed a higher level of sPD-1 and expression of PD-1 on CD4+T cell in female patients than in male patients; we identified that serum sPD-1 level and the expression of mPD-1 on T cells were significantly reduced in NSCLC; we also found that serum testosterone level increased in female patients compared with control subjects and that increased testosterone downregulated the expression of mPD-1 on T cell. These findings provide a better understanding of the differences in PD-1 expression between genders in NSCLC patients and the effect of sex hormones on PD-1 expression and supply evidence for early lung cancer diagnosis and responsiveness to immune checkpoint inhibitors.

## Introduction

Lung cancer is a leading cause of cancer-related deaths worldwide and is one of the most frequently diagnosed cancers. About 1.8 million lung cancer cases were diagnosed, and 1.2 million people died from lung cancer around the world (1–3). Growing evidence show that the lung cancer incidence in female patients continues to rise and that women have a higher risk to develop adenocarcinoma than men. These observations suggest that sex and gender are relevant to the pathogenesis of lung cancer (4, 5).

Male and female individuals differ in their innate and adaptive immune responses. Human studies demonstrate that male sex with low immune responses is more vulnerable to malignancy and infection disease than female subjects (6, 7). Even the treatment of tumors shows gender difference. Immune checkpoint inhibitors (ICIs) including programmed cell death 1 (PD1)/programmed cell death 1 ligand 1 (PD-L1)-specific monoclonal antibodies, seem to be more effective for female patients compared with male patients with melanoma (8).

The PD-1 (CD279)/PD-L1 pathway, as one of the B7 molecular family members, provides negative signals to suppress and control T cell responses. Through inducing T cell tolerance, exhaustion, and enhancing Treg cell function, the PD-1/PD-L1 pathway mediates tumor immune escape (9). High expression levels of inhibitor molecular B7 on various types of human tumor cell have been identified (10). In non-small cell lung cancer (NSCLC), fewer T cells are observed both in PD-L1+ and PD-L1 high tumor region, and a high expression level of PD-L1 on tumor cells is significantly associated with poor differentiation of tumor and poor prognosis (11–13). Soluble PD-L1 (sPD-L1) in serum, a valuable biomarker in multiple cancers, has been reported, including multiple myeloma (14), renal cell carcinoma (15), B cell lymphoma (16), and malignant melanoma (11, 17). High levels of sPD-L1 in plasma were associated with poor outcome in lung cancer with advanced stage (18, 19).

PD-1 is a type I transmembrane protein which is composed of one immunoglobulin (Ig) superfamily domain, a transmembrane domain, and an intracellular domain with immunoreceptor tyrosine-based inhibitory motif and an immunoreceptor tyrosine-based switch motif (9). PD-1 expression is induced on activated T cells, NK cells, and B cells and highly expressed on Treg cells. The major physiological biology of PD-1 is to control the infection response and autoimmunity (9, 20). An increased level of PD-1 on lymphocytes in metastatic lymph nodes from non-small cell lung cancer (NSCLC) is observed (21). PD-1 is highly expressed on Treg cells among CD4+ tumor infiltrate lymphocytes (TILs) and anergic or exhausted CD8+TILs from different multiple tumor cells, which downregulate T cell immune response (22). Exon 3 only PD-1 (PD-1dealtex3) splice variants (soluble PD-1 and sPD-1) which lack the transmembrane domain play a key in autoimmunity (23) and rheumatoid arthritis (24, 25). Increased amounts of sPD-1 can be detected in melanoma and renal cell carcinoma (14, 15). Another clinical study has shown that lung cancer patients with an increased sPD-1 have a more favorable outcome during erlotinib treatment (16).

The expression level of PD-1 (mPD-1 and sPD-1) in the sex of NSCLC and whether it can be a biomarker for the treatment with ICIs or diagnosis are both still poorly understood. In this study, we investigated the decreased serum sPD-1 level in NSCLC patients and the higher level of sPD-1 in female patients than in male patients. Similarly, there was a higher expression of PD-1 on CD4+T cell in women than in men. We also observed increased testosterone level in female patients compared with the control, and the increased testosterone downregulated the mPD-1 expression on T cells. These results revealed the sex differences in serum sPD-1, mPD-1 expression on T cells, and sex hormone levels in NSCLC patients. These findings provide a better understanding of the differences in PD-1 expression between genders in NSCLC patients and the effect of sex hormones on PD-1 expression and supply evidence for early lung cancer diagnosis and responsiveness to ICIs.

## Results

### Serum sPD-1 level was significantly reduced in patients with lung cancer

**Figure 1:**
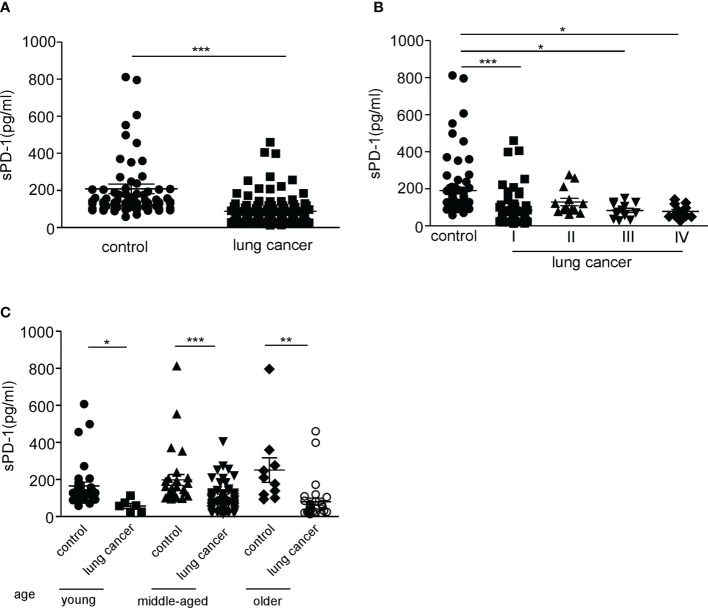
Serum sPD-1 level significantly reduced in lung cancer patients. **(A)** The serum concentration of soluble PD-1 (pg/ml) was analyzed in non-small cell lung cancer (NSCLC) patients and healthy donor groups by ELISA. **(B)** The serum levels of sPD-1 (pg/ml) were detected in clinical stage I, stage II, stage III, and stage IV from NSCLC patients by ELISA. **(C)** The sPD-1 serum levels were analyzed at different age groups of NSCLC patients and control. Young: 22–40, middle-aged: 41–64, older: 65+ years old. *P < 0.05, **P < 0.005, ***P < 0.0001. Mann–Whitney test (two-tailed) and non-paired Student’s *t*-test.

To identify the level of sPD-1 in serum from NSCLC patients, we recruited 108 lung cancer patients and 72 healthy donors. No significant differences were detected in their age distributions (**Supplementary Figures S1A, B** **)**. We tested the level of sPD-1 in the serum of lung cancer patients, and our results showed that the serum sPD-1 level (87.99 ± 7.568, *N* = 108) of lung cancer patients was significantly lower than that of the control group (190 ± 17.68, *N* = 72) (**Figure 1A**, ***P < 0.0001); no difference was observed of serum sPD-1 in all stages of NSCLC, while downregulation of sPD-1 was found in early-stage NSCLC (82.98 ± 10.43, *N* = 71) (**Figure 1B**, ***P < 0.0001). Moreover, the sPD-1 levels were significantly lower in stage II (128.9 ± 19.96, *N* = 13) and stage III (83.28 ± 11.13, *N* = 13) than in the control group (**Figure 1B**, *P < 0.05).We found a difference in age between the control group and the lung cancer patients; the age of the lung cancer group (57.40 ± 1.009, *N* = 108) was significantly higher than that of the control group (44.65 ± 1.574, *N* = 72) (**Supplementary Figure S1C**). To determine whether the difference in sPD-1 was due to age, we tested the serum levels of sPD-1 at different age groups of the control. The level of sPD-1 in the older subjects (251.0 ± 66.20, *N* = 10) tended to be higher than those of the young subjects (165.7 ± 20.77, *N* = 34) and middle-aged group (197.8 ± 29.57, *N* = 28), but there was no statistical difference; similar results were observed in lung cancer patients (**Figure 1C**). The serum sPD-1 levels were significantly decreased in lung cancer patients at different age groups than that of the control (**Figure 1C**, *P < 0.05, **P < 0.005, ***P < 0.0001). These results indicated that the serum sPD-1 of lung cancer patients was significantly lower than that of the control group regardless of age.

### Higher level of serum sPD-1 in female lung cancer patients than in male patients

**Figure 2:**
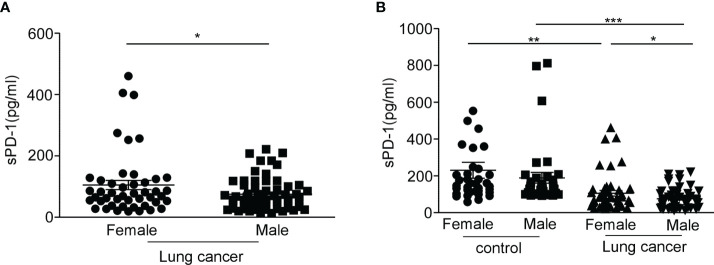
The level of serum sPD-1 in female lung cancer patients is higher than that in male patients. Serum sPD-1 levels were examined by ELISA. **(A)** The concentration of soluble PD-1 in serum (pg/ml) was analyzed in female and male non-small cell lung cancer patients. **(B)** The serum levels of sPD-1 (pg/ml) were detected in female and male controls. *P < 0.05, **P < 0.005, ***P < 0.0001; Mann–Whitney test (two-tailed) and non-paired Student’s *t*-test.

To clarify whether the level of sPD-1 differs between the sexes, we detected the serum sPD-1 expression in male and female lung cancer patients. The results showed that the serum sPD-1 level of female patients (105.3 ± 15.14, *N* = 46) was significantly higher than that of male patients (75.17 ± 6.571, *N* = 62) (**Figure 2A**, *P = 0.048). There was no difference in age between male and female patients (**Supplementary Figure S1A**). There was no difference in the serum sPD-1 levels between male and female individuals in the control group. The serum levels in both male (***P < 0.001) and female patients (*P* < 0.005, **P = 0.0041) with lung cancer were lower than those in the control group (female control, 191.0 ± 20.87, *N* = 35; male control,189.1 ± 28.48, *N* = 37) (**Figure 2B**). These results indicated that there was a difference in the serum sPD-1 level between different genders in lung cancer.

### Higher expression of membrane PD-1 on CD4+T cell in female lung cancer patients than that of male patients

**Figure 3:**
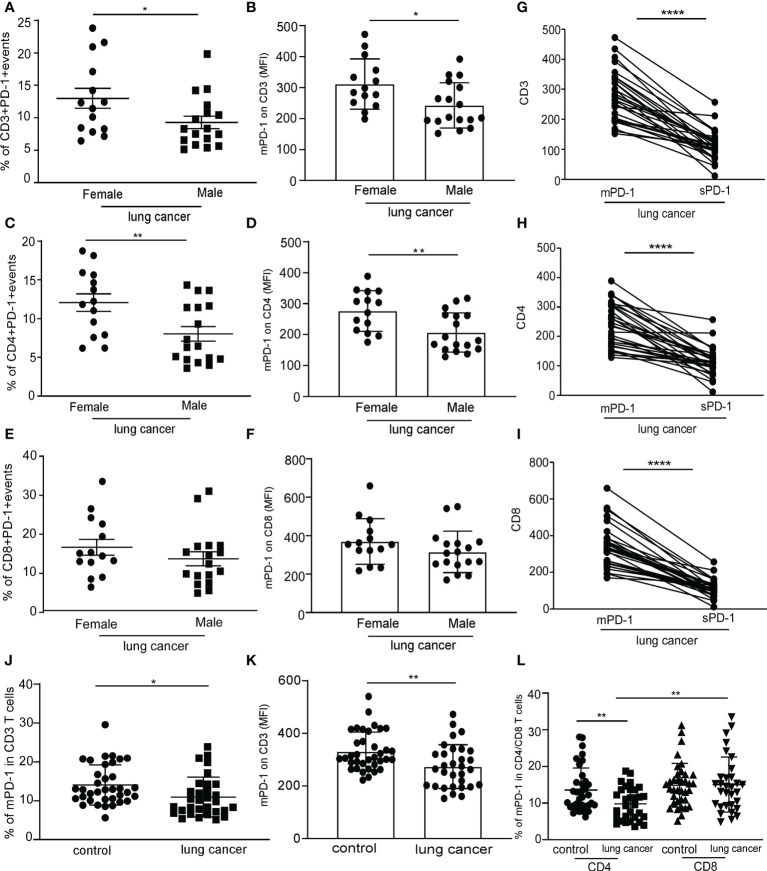
Higher expression of PD-1 on CD4+T cell in female lung cancer patients than that in male patients. Peripheral blood mononuclear cells (PBMCs) were separated from the blood of female and male lung cancer patients and were analyzed by flow cytometry; frequency of the CD3+PD-1+T cell **(A)**, CD4+PD-1+T cell **(C)**, and CD8+PD-1+T cell **(E)** in PBMCs from human lung cancer patients, expressed as mean ± SEM. *P < 0.05, **P < 0.01; Mann–Whitney test (two-tailed) and nonpaired Student’s *t*-test. Mean fluorescence intensity (MFI) of the CD3+PD-1+T cell **(B)**, CD4+PD-1+T cell **(D)**, and CD8+PD-1+T cell **(F)** in PBMCs from human lung cancer patients, expressed as mean ± SEM. *P < 0.05, Mann–Whitney test (two-tailed) and nonpaired Student’s *t*-test; ligature between the expression of mPD-1 on CD3+T cell **(G)**, CD4+T cell **(H)**, CD8+T cell **(I)**, and serum sPD-1 on individual from lung cancer patients. **(J)** Percentage of CD3+PD-1+T cells in PBMCs from control and lung cancer patients. **(K)** MFI of CD3+PD-1+T cells in PBMCs from control and lung cancer patients. **(L)** Percentage of CD4+/CD8+PD-1+T cells in PBMCs from control and lung cancer patients, expressed as mean ± SEM. *P < 0.05, **P < 0.01, ****P < 0.0001, Mann–Whitney test (two-tailed) and nonpaired Student’s *t*-test.

In the studies mentioned above, the level of sPD-1 in the serum of female lung cancer patients was higher than that of male patients. To determine whether the mPD-1 expression level was also higher in female than in male lung cancer patients, we examined the expression of mPD-1 on T cells from peripheral blood mononuclear cells (PBMCs). We found that the membrane PD-1 was substantially higher in frequency (**Figure 3A**, *P = 0.0431) and MFI (**Figure 3B**, *P = 0.0186) on CD3+T cells from female patients compared with those derived from male patients. A similar result was obtained on CD4+T cells (**Figure 3C**, **P = 0.0093, **Figure 3D**, **P = 0.0056). We did not detect a significant difference in the expression of mPD-1 on CD8+T cells between men and women (**Figures 3E, F**). CD3+/CD4+/CD8+T cells derived from NSCLC patients had a higher mPD-1, and there was a higher level of sPD-1 in serum (**Figures 3G–I**, ****P < 0.0001). These results suggest that mPD-1 might be the source of sPD-1 in lung cancer. Meanwhile, in the control, we did not observe a difference of mPD-1 expression in CD3+T cells (**Supplementary Figure S3A**), CD4+T cells (**Supplementary Figure S3B**), and CD8+T cell (**Supplementary Figure S3C**) between men and women. By the way, the percentage of CD8+ PD-1+T cells was higher than that of CD4+PD-1+T cells in lung cancer patients (**Figure 3L**, ***P = 0.0001). In addition, we observed that the expression of mPD-1 on T cells in lung cancer patients was significantly lower than that in the control group (**Figure 3J**, *P = 0.0166; **Figure 3K**, **P = 0.0039). The frequency of PD-1on CD4+T cells of lung cancer patients was lower than that of the control group (**Figure 3L**, **P = 0.006). We also analyzed the MFI of PD-1 on T cells in the control group and lung cancer groups at different ages. There was no difference between lung cancer patients of different ages, and similar results were found in the control group (**Supplementary Figures S3D–I**), although the men in the control group were older than the women (**Supplementary Figure S3J**, *P = 0.0166).

These results indicated that the expression of mPD-1 on T cells (especially on CD4+T cell) in lung cancer patients was consistent with that of serum sPD-1. It was different between female and male patients, and the expression of mPD-1 on T cells was not affected by age.

### Serum testosterone level increased in female lung cancer patients

**Figure 4:**
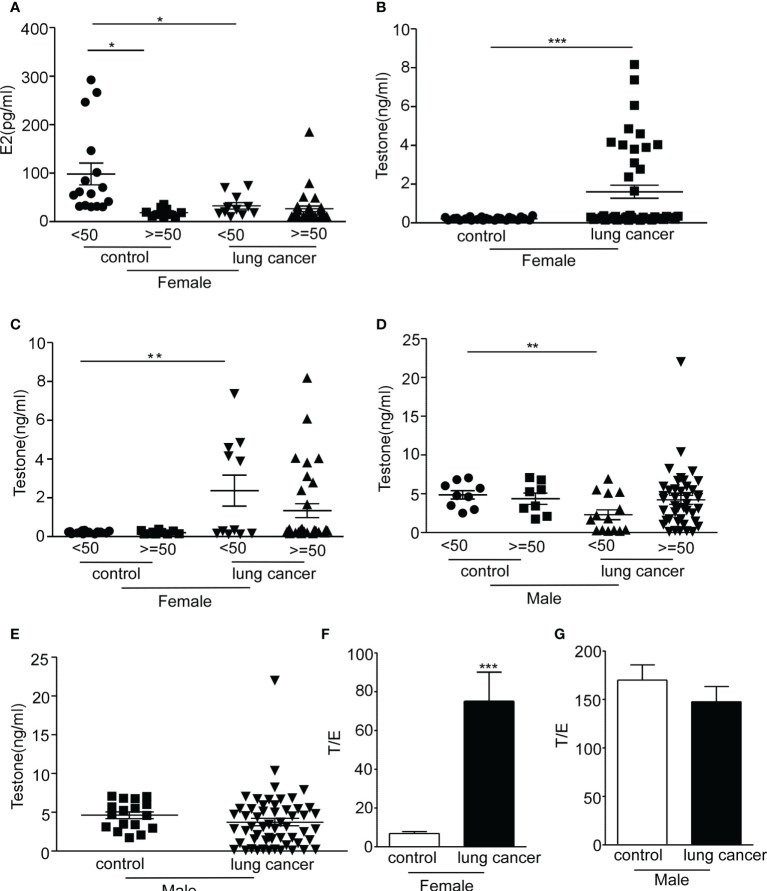
Serum concentration of sex hormone in lung cancer patients. The serum estradiol and testosterone levels were examined by CMIA. **(A)** The concentration of estradiol in serum (pg/ml) was analyzed in female non-small cell lung cancer (NSCLC) patients and control at different ages. **(B)** The serum levels of testosterone (ng/ml) were detected in female NSCLC patients and control. **(C)** The serum concentration of testosterone (ng/ml) was analyzed in female NSCLC patients and control at different ages. **(D)** The concentration of estradiol in serum (pg/ml) was analyzed in male NSCLC patients and control at different ages. **(E)** The serum concentration of testosterone (ng/ml) was examined in male NSCLC patients and control. **(F)** Ratio of testosterone to estradiol (T/E) in women with lung cancer and control. **(G)** Ratio of testosterone to estradiol (T/E) in men with lung cancer and control. *P < 0.05, **P < 0.005, ***P < 0.0001; Mann–Whitney test (two-tailed) and non-paired Student’s *t*-test. E2, estrogen; T/E, androgen/estrogen.

The data mentioned above indicate that the expression of serum sPD-1 and mPD-1 of female patients was higher than that of male patients. Whether this is due to the sex hormone remains uncertain. Serum estrogen decreases with age and declines substantially in menopause, which occurs at the age of 51 years (26). We first measured the serum levels of estradiol in female patients. No significant difference was observed in estrogen level between the age group over 50 years old (26.35 ± 5.883, *N* = 31) and the age group under 50 years old (32.55 ± 6.715, *N* = 11); in the control group, the serum estradiol levels were consistent with the reports (26) (**Figure 4A**, *P = 0.0106). Our results also showed that NSCLC patients lack a gender difference in serum estradiol (**Supplementary Figure S3A**). Compared with the control group, no difference in estradiol levels was observed in male NSCLC patients at different ages (**Supplementary Figure S3B**). In addition, the testosterone level in female NSCLC patients (1.265 ± 0.2931, *N* = 39) was approximately fivefold higher than those in the control group (0.2566 ± 0.02916, *N* = 41) (**Figure 4B**, ***P = 0.0007). The serum concentration of testosterone in female patients under 50 years old was higher than those in the control group (**Figure 4C**, **P = 0.003). Conversely, there was a lower testosterone level in male patients under 50 years old than those in the control group (**Figure 4D**, *P = 0.0105). No difference could be demonstrated in the testosterone levels among male patients of different ages nor in the control group (**Figures 4D, E** **)**, except that the testosterone level of male patients was significantly higher than that of female patients (**Supplementary Figure S3C**, ***P = 0.001). Based on the results of the expression levels of estradiol and testosterone in lung cancer, we found that the ratio of testosterone to estradiol (T/E) in women was significantly higher due to the higher levels of serum testosterone compared with the control (**Figure 4F**, ***P = 0.0007). There is no significant change to the T/E ratio in men with lung cancer in comparison with the control (**Figure 4G**). These observations revealed that the sex hormone is remarkably changed in the serum from female lung cancer patients.

### Testosterone downregulates PD-1 expression on T cells

**Figure 5:**
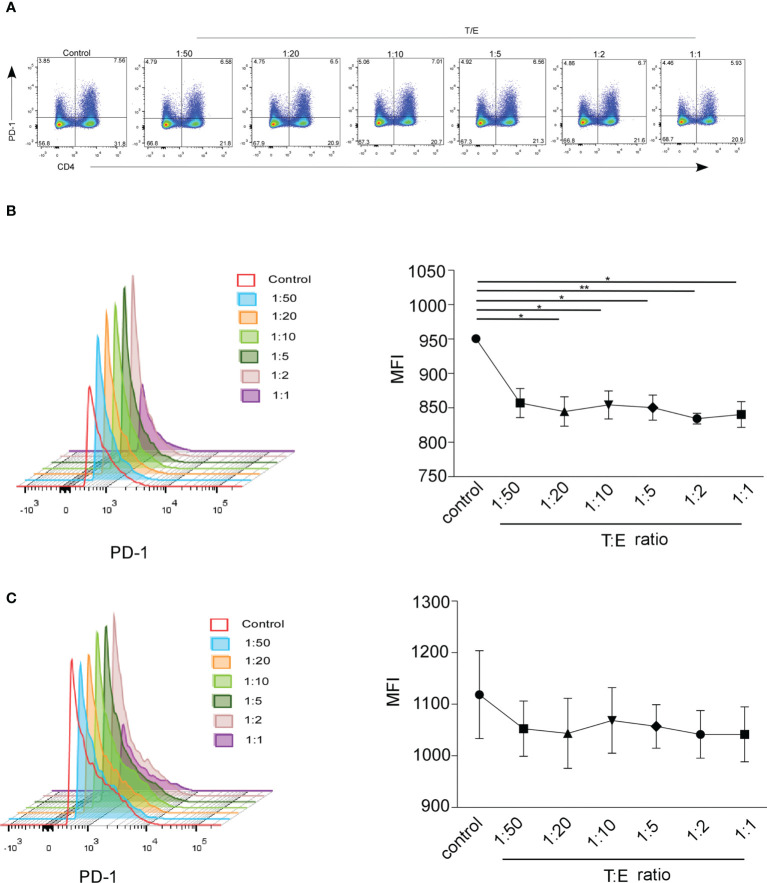
The MFI of mPD-1+ CD4+/CD8+T cells from healthy control was analyzed by FACS. Peripheral blood mononuclear cells (PBMCs) were treated 24 h with different concentrations of estrogen (10, 20, and 50 nmol) and androgen (1 and 10 nmol). **(A)** Frequency of CD4+/CD8-PD-1+ cells and CD4-/CD8+PD-1+ cells which gated on CD3+T cells treated with the ratio of T/E at 1:50 (1 nmol androgen and 50 nmol estrogen), 1:20 (1 nmol androgen and 20 nmol estrogen), 1:10 (1 nmol androgen and 10 nmol estrogen), 1:5 (1 nmol androgen and 5 nmol estrogen); 1:2 (1 nmol androgen and 2 nmol estrogen), and 1:1 (1 nmol androgen and 1 nmol estrogen) (healthy donor, *n* = 3). The MFI of CD8+PD-1+T cell **(B)** and CD4+PD-1+T cell **(C)** in PBMCs with the ratio of T/E at 1:50, 1:20, 1:10, 1:5, 1:2, and 1:1. *P < 0.05, **P < 0.01. Mann–Whitney test (two-tailed) and paired Student’s *t*-test. T/E, androgen/estrogen; MFI, mean fluorescence intensity.

Multiple studies observed that sex hormones regulate the expression of PD-1. Estrogen administration increased the intracellular PD1 expression in CD4+FoxP3+T cells, and PD1 expression was reduced in ER knockout mice (23, 27–29). The expression of PD-1 regulated by androgen has not been extensively explored. Low serum sPD-1 and high testosterone circulating levels were observed in NSCLC female patients. To investigate PD-1 expression on T cells in response to different concentrations of testosterone and estrogen *in vitro*, we isolated PBMCs from healthy donors, cultured them with hormones or phosphate-buffered saline for 24 h, and detected the percentage and MFI of PD-1+CD4+T cells and CD8+T cells by FACS. In the representative flow cytometric analysis on CD4+/CD8-PD-1+, CD4-/CD8+PD-1+ cells which gated on CD3+T cells (**Figure 5A**), the frequency of CD8+PD-1+ cells increased after testosterone and estrogen treatment with T/E ratios of 1:50, 1:20, 1:10, and 1:5 (**Supplementary Figure S4A**), whereas we found that the MFI of PD-1 on CD8+T cells decreased when given the testosterone and estrogen treatment, but no direct association between the dose of treatment and downregulation of PD-1 had been observed (**Figure 5B**, *P < 0.05, **P < 0.01). The MFI of PD-1 on CD4+T cells was decreased after application of the testosterone and estrogen treatment (**Figure 5C**). The frequency of CD4+PD-1+T cells was significantly reduced with the 10 nM androgen and 20 nM estrogen treatment (**Supplementary Figure S4B**, *P < 0.05). These results indicated that the increased level of testosterone could inhibit the expression of PD-1 on T cells, especially on CD8+T cells.

### Reduced expression of estrogen receptor/androgen receptor on T cells in lung cancer patients

**Figure 6:**
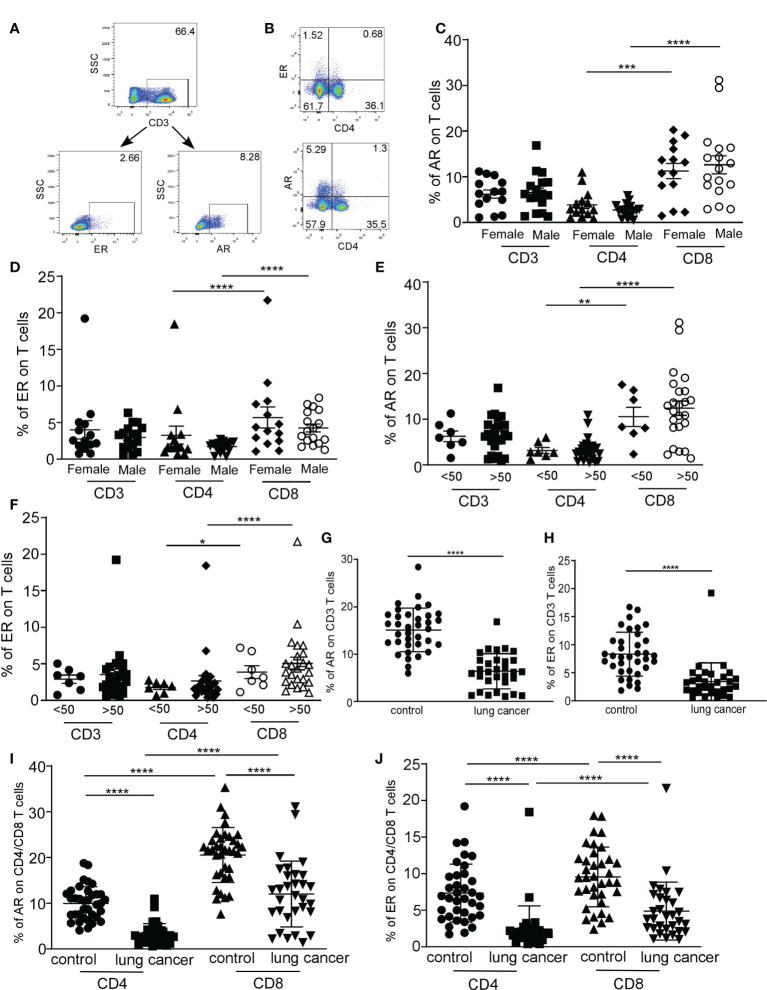
The distribution of estrogen receptor (ER) and androgen receptor (AR) on T cells in lung cancer patients was analyzed by FACS. Representative flow cytometric analyses of AR and ER expression on CD3 T cells **(A)** and representative flow cytometric analyses of CD4+/CD8-ER+, CD8+/CD4-ER+T cells (top quadrant), CD4+/CD8-AR+, and CD8+/CD4-AR+T cells (bottom quadrant), gated on the CD3 populations **(B)**. The percentage of AR expression on T cells **(C)** and ER expression on T cells **(D)** in CD3, CD4, and CD8 T cells from female and male lung cancer patients. The percentage of AR expression on T cells **(E)** and ER expression on T cells **(F)** in CD3, CD4, and CD8 T cells from lung cancer patients at different ages. The percentage of AR **(G)** and ER **(H)** expression on CD3+T cells from lung cancer patients and control. The percentage of AR **(I)** and ER **(J)** expression on CD4/CD8+T cells from lung cancer patients and control. ***P < 0.001, ****P < 0.0001; Mann–Whitney test (two-tailed) and nonpaired Student’s *t*-test. * P<0.05, **P<0.01.

Hormones play physiological functions by binding to their receptors. It has been known that an estrogen receptor (ER) was found in T cell subsets (30). Studies have demonstrated that the androgen receptor (AR) was expressed on lymphocytes (31). Very little is known about whether the expressions of ERs and ARs vary between lung cancer patients and control subjects. We detected the expression of AR and ER on CD3+T, CD4+T, and CD8+T cells in lung cancer patients, and there was no difference between men and women (**Figures 6A, B**). Both in female and male patients, CD8+T cells had a higher expression of AR and ER, corresponding to CD4+T cells (**Figures 6C, D**, ***P < 0.0001). No difference in the expression of hormone receptors on T cells in lung cancer patients was observed between under those who were 50 years old and over 50 years old (**Figures 6E, F**). These results showed that the expression of hormone receptors on the T cells was regardless of gender and age. AR and ER were substantially lower in frequency on CD3+T cells and CD4+/CD8+T cells from NSCLC patients compared with those derived from the control group, and a higher expression of AR and ER on CD8+T cells than that of CD4+T cells was observed in the control and lung cancer patients (**Figures 6G–J**, ****P < 0.0001). The results indicated that the expression of hormone receptors on the surface of CD4+T cells and CD8+T cells presents a distinct pattern.

## Correlation analysis of expression of mPD-1 and ER/AR on T cells of lung cancer patients

We observed that the ER and AR expressions in NSCLC are higher in CD8+T cells than in CD4+T cells. Similarly, mPD-1 expression is higher in CD8+T cells than in CD4+T cells. To investigate the effect of the expression of hormone receptors and mPD-1 which were derived from CD3, CD4, and CD8T cells, we performed a linear regression analysis between the frequency of hormone receptors and mPD-1. ER expression did not correlate with mPD-1 on CD3+, CD4+, and CD8+T cells in NSCLC patients (**Supplementary Figure S5A**); however, ER shows a positive correlation with mPD-1 on CD3+T cells (**Supplementary Figure S5A**, *R* ^2^ = 0.376, ****P < 0.0001), CD4+T cells (**Supplementary Figure S5A**, *R* ^2^ = 0.3996, ****P < 0.0001), and CD8+T cells (**Supplementary Figure S5A**, *R* ^2^ = 0.265, **P = 0.0013) from the healthy control. Similar results show that the expression of AR and mPD-1 on T cells was not correlated in NSCLC patients (**Supplementary Figure S5B**), and that of AR was correlated with mPD-1 on CD4+T cells from the healthy control. This correlation is significant (**Supplementary Figure S5B**, *R* ^2^ = 0.1374, *P = 0.03). Next, we investigated the correlation of ER and AR. Significantly positive correlations were observed between AR and ER on CD3+T cells (**Supplementary Figure S5C**, *R* ^2^ = 0.2908, **P = 0.0017, CD4+T cells (**Supplementary Figure S5C**, *R* ^2^ = 0.5356, ****P < 0.0001) and CD8+T cells (**Supplementary Figure S5C**, *R* ^2^ = 0.2812, **P = 0.0022) from NSCLC patients. Similar findings were investigated in the control group, such as (1) AR and ER on CD3+T cells (**Supplementary Figure S5C**, *R* ^2^ = 0.4077, ****P < 0.0001), (2) AR and ER on CD4+T cells (**Supplementary Figure S5C**, *R* ^2^ = 0.6476, ****P < 0.0001), and (3) AR and ER on CD8+T cells (**Supplementary Figure S5C**, *R* ^2^ = 0.3234, ***P = 0.0003). These results altogether demonstrated that hormone receptors do not correlate with mPD-1 in NSCLC patients; these results also demonstrated the positive correlation between ER and AR.

## Discussion

The purpose of our study was to investigate the expression level of PD-1 (including PD-1 in serum and membrane PD-1 on T cells) in NSCLC between male and female patients. We found that the serum sPD-1 from NSCLC patients is significantly decreased compared with that of tumor-free individuals, especially in phase I of NSCLC patients, suggesting that sPD-1 can serve as a potential marker for the early diagnosis of lung cancer.

Different studies have shown that the expression of soluble PD-1 in the serum is inconsistent in various tumor types. sPD-1 level is increased in melanoma and renal carcinoma (22), but our studies demonstrated that the expression level of serum sPD-1 was decreased in NSCLC. Two reasons could explain the decline of sPD-1; one of the reasons may be related to the binding of the ligand to PD-L1. The expression of membrane-bound PD-L1 which was present on the surface of tumor cells and sPD-L1 in the serum of patients with NSCLC was increased and associated with lung cancer TNM staging and prognosis (32, 33). PD-L1 has been used as a biomarker for lung cancer diagnosis. In NSCLC patients, the expression of PD-L1 increased on tumor cells. The sPD-1 in serum combined with PD-L1, which then led to a decline of detectable serum sPD-1. From this point of view, our study is consistent with Steffen’s research, whose report indicated that increased sPD-1 was associated with better prognosis with advanced EGFR-mutated NSCLC patients treated with erlotinib (24).

Another reason may be related to the reduction of sPD-1 production. At present, the source of sPD-1 is still unclear. PD-1 is encoded by pdcd1gene, and sPD-1 belongs to the PD-1dealt ex3 splice variant and lacks the transmembrane domain. The downregulation of pdcd1 gene could lead to the decreased expression of sPD-1 and mPD-1. We observed that the expression of mPD-1 on T cells and CD4+T cells in lung cancer patients was significantly lower than that in the control group (**Figure 3L**), although a definitive positive correlation between the serum sPD-1 level and MFI of mPD-1 on T cells was not observed. We found that both of them show a consistent trend of change. NSCLC patients with high serum sPD-1 levels also have a high expression of mPD-1 on T cells. Patients with low serum sPD-1 levels also have a relatively low expression of mPD-1on T cells. Further work is needed to reveal the relationship between serum sPD-1 level and mPD-1 expression on T cells in NSCLC patients with immune checkpoint blockade (ICB) treatment. We collected the NSCLC samples of those who were treated with anti-PD-L1 or anti-PD-1 immunotherapy and observed the changes in the level of sPD-1 and mPD-1 that would be direct clinical evidence for evaluating the response to ICB and exploring the application in drug therapy.

Our study also investigated that the level of sPD-1 was reduced in male patients compared with that in female patients. There was no difference in the estrogen levels between men and women with lung cancer. The testosterone level of male patients was significantly higher than that of female patients. We also observed a higher testosterone level in female patients than in control subjects, while a lower serum sPD-1 level was found in female patients than in the control. Due to the older age of onset of tumor patients compared with the control and in order to make our laboratory research meaningful to clinical, in further research, we expanded the samples of NSCLC in female patients and compared the expression level of testosterone and sPD-1 in the same age group with the control group. In our study, although we cannot observe the direct inhibitory effect of testosterone on sPD-1 expression, as we have discussed before, the changes of sPD-1 and mPD-1 are consistent. Considering that estrogen and androgens are present in the body of both men and women, we treated the PBMCs from healthy donors with testosterone and estrogen but gradually increase the concentration of testosterone. The mPD-1 expression on T cells was inhibited without being dose dependent. In general, androgens have immunosuppressive roles on the immune responses. Testosterone increased the Tregs expansion and TGF-β production, and the negative selection of thymocytes promotes central self-tolerance. Testosterone inhibits Th1 differentiation and cytokine production (IL-12 and IFN-γ). Testosterone also causes the death of effector T cells, limits the peripheral lymphocyte compartment, and suppresses the inflammation responses (34), but the mechanism of testosterone’s inhibition of PD-1 expression needs to be further explored.

Sex hormone mediates the physiological role by ligand–receptor interaction. Immune cells express sex hormone receptors and respond to sex hormones (35). Researchers reported that suppressor/cytotoxic lymphocytes express ERs, while helper lymphocytes were found in the absence of receptors for steroid hormones (36, 37). There are very limited reports on the difference in AR expression among different T cell subsets. Our research found that gender and age did not affect the expression of the hormone receptors (AR and ER) on the surface of T cells. The expression of AR and ER on CD8 T cells is higher than that on CD4 T cells. We also observed that both the frequency of CD8+PD-1+T cell and the MFI of PD-1on CD8+T cell are higher than those of CD4+T cells in NSCLC. Our results showed that positive a correlation was observed between the expression of hormone receptors and mPD-1 both on T cells from the control subjects, but in lung cancer patients, no statistically significant correlation was detected. Meanwhile, an inverse correlation trend was observed between the expression of ER and PD-1 on CD3 T cells in NSCLC. A study reported that high estrogen receptor downregulated the expression PD-1/PD-L1 in breast cancer, thus leading to the reduction of CD8+T cells infiltrating the tumor by inhibiting the IL-17 signal transduction (38). Whether there is a similar mechanism for PD-1 expression regulated by ER on different cells requires further research. Another question worth discussing is the positive correlation between the expression of ER and PD-1 on T cells in the control group. Furthermore, we found a positive correlation between the expression of AR and ER on T cells both in NSCLC patients and control subjects. It illustrates that the expression level of PD-1 in NSCLC patients has changed a lot, which is likely to be mediated by the hormone and hormone receptor interactions.

In conclusion, our study demonstrated that the serum sPD-1 level was significantly reduced in NSCLC patients and that a higher level of serum sPD-1 was found in female patients than in male patients, and the increased testosterone level downregulated the expression of mPD-1 on T cells. This finding provides a better understanding of the differences in PD-1 expression between genders in NSCLC patients and the effect of sex hormones on PD-1 expression and supplies evidence for early lung cancer diagnosis and responsiveness to ICB.

## Materials and methods

### Ethics statement

Written informed consent was obtained from all patients and tumor-free donors. This study was approved by the ethics committees of the Zhong Shan School of Medicine, Sun Yat-Sen University (Guangzhou, China), and the First Affiliated Hospital of Sun Yat-Sen University (Guangzhou, China).

### Study participants

In total, 108 NSCLC patients were enrolled from the First Affiliated Hospital of Sun Yet-San University of Guang Zhou, China, with 46 female and 62 male patients, and their ages ranged from 28 to 83 years old: six young patients (ages 22–40: four men and two women), 72 middle-aged patients (ages 41–64: 39 men and 33 women), and 30 older subjects (65+: 19 men and 11 women). The diagnosis of lung cancer was based on pathological evidence (detected by histological staining), with phase I (73 cases), phase II (13 cases), phase III (13 cases), and phase IV (11 cases) (shown in **Table 1**). Patients whose serology tested positive for HIV, HBV, and HCV and those who were free of other tumors were excluded from the study. None of the patients received cancer-related chemotherapy during the period of collecting the samples. In total, 72 tumor-free donors were recruited for blood collection (with age range from 21 to 82 years), with a total of 37 female and 35 male individuals (shown in **Table 2**). Those whose serology tested positive for HIV, HBV, and HCV and those who were free of tumors were excluded from the study.

**Table 1:** Non-small cell lung cancer patient samples.

Age	NSCLC-patients (n%)	P value	tumor Free (n%)	P value
22-40 (young age)	6 (5.5)	0.47 ( one-way ANOVA	34 (17)	0.27 ( one-way ANOVA
41-64 (middle-age)	72 (66.7)	and nonparametric)	28 (38.9)	and nonparametric)
>=65 (old age)	30 (27.8)		10 (13.9)	
**Stage**				
I	71 (65.7)	0.12 ( one-way ANOVA		
II	13 (12)	and nonparametric)		
III	13 (12)			
IV	11 (10.2)			
**Gender**				
Male	62 (57.4)	0.92	35 (48.6)	0.54
Female	46 (42.6)		37 (51.4)	

**Table 2:** Healthy donor samples.

**Feature**	**Cases**
Age	
22-40	34
41-64	28
65+	10
Gender	
Male	35
Female	37

### Blood samples

Blood was collected intravenously from each participant. The samples were centrifuged at 3,000 rpm for 10 min at 4°C. The supernatant was transferred to Eppendorf tubes and centrifuged at 12,000 rpm for 10 min at 4°C. The serum was isolated and stored at −80°C for subsequent ELISA assays. All patients had pathologist-confirmed NSCLC and adenocarcinoma or adeno-squamous histologic subtype and stage according to WHO classification 200438.

### Soluble PD-1 ELISA

A commercial ELISA kit (Thermo, catalog EHPDCD1) was used to test the sPD-1 concentration in 100 ul serum without dilution. The assay procedures were performed according to the manufacturer’s instructions. The concentration of soluble PD-1 was determined by the mean optical density of 450 nm of the two duplicates analyzed, and a standard curve was fitted with four-parameter logistic regression.

### Serum estradiol and testosterone CMIA

A commercial Estradiol Reagent Kit (Abbott, ref. 7K72/07P50) and a commercial 2nd Generation Testosterone Reagent Kit (Abbott, ref. 2P13/07P68) were used to test the estradiol/testosterone concentration in the serum. The assay procedures were performed according to the manufacturer’s instructions. Acridinium ester, as a label, was combined with estradiol/testosterone. In the reaction system, the chemiluminescence reaction produced by the acridine ester was measured and expressed in relative luminescence units (RLUs). There is an inverse relationship between the amount of estradiol/testosterone in the sample and the value of RLUs as detected by the optical system. The estradiol/testosterone concentrations were calculated from a calibration curve of known estradiol/testosterone concentrations.

### Preparation of PBMCs

PBMCs were isolated by Ficoll-Hypaque (Tian Jin Hao Yang Biological Manufacture Co., Ltd., China, catalog LTS1077) gradient centrifugation of sodium heparin–blood obtained from healthy donors or lung cancer patients.

### Flow cytometry

Fluorescently conjugated antibodies were purchased from BD Biosciences, eBioscience, TONBO biosciences, and Bioss for phenotypic analyses. The following panel of mouse anti-human mAbs was used: APC.cy7-conjugated anti-CD3 (BD Biosciences, 557832, SK7) was purchased from BD Biosciences (San Jose, CA, USA), Percp.cy5.5-conjugated anti-CD4 (TONBO biosciences, 65-0048-T100, OKT4) was purchased from TONBO biosciences, Inc. (San Diego, CA, USA), PE-conjugated anti-PD-1 (eBioscience,12-2799-42, eBioJ105) was purchased from eBioscience, Inc. (San Diego, CA, USA), and AF-488-conjugated-anti-AR (Bioss, bs-0118R-AF488) and APC-conjugated-ER (Bioss, bs-0174R-APC) were both purchased from Bioss Antibodies Inc. (Woburn, Massachusetts, USA). Estradiol valerate (Selleckchem, S4757) and dihydrotestosterone (Selleckchem, S3149) were all purchased from Selleckchem (Houston, TX, USA). The PBMCs from healthy donors and lung cancer patients were stained for flow cytometry. The cells were then harvested and stained for flow cytometric analysis. All samples were collected on FACSAria II BD (Mountain View, CA, USA). Data were analyzed using Flow Jo software (Tree Star, San Carlos, CA, USA).

### Statistics

Statistical analysis was performed on the GraphPad Prism software version 5. The Mann–Whitney test (two-tailed) and non-paired Student’s *t*-test were performed to determine the statistical differences in the two groups. A value of *P* < 0.05 was considered statistically significant. Statistical associations between groups and gender were assessed using Chi-square test. P< 0.05 means deference in statistics. The analysis software is SPSS version 20.

## Data availability statement

The original contributions presented in the study are included in the article/**Supplementary Material**. Further inquiries can be directed to the corresponding author.

## Ethics statement

The studies involving human participants were reviewed and approved by Zhongshan Medical Ethics Committee. Written informed consent for participation was not required for this study in accordance with the national legislation and the institutional requirements.

## Author contributions

YG recruited NSCLC patients, analyzed data, and contributed to manuscript preparation. YT performed experiments involving cell culture and soluble PD-1 detection and prepared the figures. JW collected serum and performed experiments involving serum estradiol and testosterone CMIA. JZ and CL recruited NSCLC patients and control subjects and analyzed data. HZ and YW prepared PBMCs, performed experiments involving FACS, and analyzed data. SS performed experiments and analyzed data. HL and JY contributed to manuscript preparation. HH conceived, designed, and supervised the experimental procedures and prepared the manuscript. All authors contributed to the article and approved the submitted version.

## Funding

This study was supported by Guangzhou Science Foundation of China (2019A1515010190).

## Acknowledgments

We thank other members of the laboratory for their assistance.

## Conflict of interest

The authors declare that the research was conducted in the absence of any commercial or financial relationships that could be construed as a potential conflict of interest.

## Publisher’s note

All claims expressed in this article are solely those of the authors and do not necessarily represent those of their affiliated organizations, or those of the publisher, the editors and the reviewers. Any product that may be evaluated in this article, or claim that may be made by its manufacturer, is not guaranteed or endorsed by the publisher.

## Supplementary material

Supplementary Figure 1 Non-small cell lung cancer (NSCLC) and control sample information. **(A)** Analysis of sexes and age distributions from NSCLC. **(B)** The analysis of sexes and age distributions from control. **(C)** Analysis of age distributions for both NSCLC and control. ****P* < 0.0001; Mann–Whitney test (two-tailed) and non-paired Student’s *t*-test.

Supplementary Figure 2 The expression of mPD-1 on T cells in lung cancer patients and control at different ages. The percentage and MFI of CD3+PD-1+T cell **(A)**, CD4+PD-1+T cell **(B)**, and CD8+PD-1+T cell **(C)** in peripheral blood mononuclear cells (PBMCs) from control. The MFI of the CD3+PD-1+T cell **(D)**, CD4+PD-1+T cell **(E)**, and CD8+PD-1+T cell **(F)** in PBMCs from human lung cancer patients in young, middle-aged, and old subjects. The MFI of the CD3+PD-1+T cell **(G)**, CD4+PD-1+T cell **(H)**, and CD8+PD-1+T cell **(I)** in PBMCs from control in groups under 40 years old and above 40 years old. **(J)** Analysis of age distribution between men and women in the control. **P* < 0.05, ****P* < 0.001, *****P* < 0.0001, Mann–Whitney test (two-tailed) and nonpaired Student’s *t*-test. Young, 21–40 years old age group; middle-aged, 41–64 years old age group; old subjects, 65+ years old; LC, lung cancer; MFI, mean fluorescence intensity.

Supplementary Figure 3 Serum concentration of estradiol and testosterone in lung cancer patients. The serum estradiol and testosterone levels were examined by CMIA. **(A)** The serum concentration of estradiol (pg/ml) was analyzed in non-small cell lung cancer (NSCLC) patients. **(B)** The serum levels of estradiol (pg/ml) were detected in male NSCLC patients and control at different ages. **(C)** The serum concentration of testosterone (ng/ml) was analyzed in NSCLC patients. ****P* < 0.0001; Mann–Whitney test (two-tailed) and non-paired Student’s *t*-test. E2, estradiol.

Supplementary Figure 4 The percentage of mPD-1+ CD4+/CD8+T cells from healthy control was analyzed by FACS. Peripheral blood mononuclear cells (PBMCs) were treated for 24 h with different concentrations of estrogen (10, 20, and 50 nmol) and androgen (1 and 10 nmol). **(A)** The frequency of CD8+PD-1+T cells **(A)** and CD4+PD-1+T cells **(B)** which were treated with the ratio of T/E at 1:50 (1 nmol androgen and 50 nmol estrogen), 1:20 (1 nmol androgen and 20 nmol estrogen), 1:10 (1 nmol androgen and 10 nmol estrogen), 1:5 (1 nmol androgen and 5 nmol estrogen), 1:2 (1 nmol androgen and 2 nmol estrogen), and 1:1 (1 nmol androgen and 1 nmol estrogen) (healthy donor, *n* = 3). **P* < 0.05, Mann–Whitney test (two-tailed) and paired Student *t*-test. T/E, androgen/estrogen.

Supplementary Figure 5 Correlation analysis of the expression of mPD-1 and ER/AR on T cells. **(A)** Correlation between the expression of ER and mPD-1 on CD3 T cells, CD4 T cells, and CD8 T cells from non-small cell lung cancer (NSCLC) and control subjects. **(B)** Correlation between the expression of AR and mPD-1 on CD3 T cells, CD4 T cells, and CD8 T cells from NSCLC and control subjects. **(C)** Correlation between the expression of ER and AR on CD3 T cells, CD4 T cells, and CD8 T cells from NSCLC and control subjects. *P*-values were obtained by two-tailed Spearman’s test. Linear regression analysis was performed in GraphPad Prism to get the *R* ^2^ and *P*-values. *R* ^2^, goodness of fit; *P*, significance of the slope deviation from zero; NS, non-significant (*n* = 36).
