# Supplementary figures and images for: Sex difference in the expression of PD-1 of non-small cell lung cancer
Source: Front Immunol. 2022 Oct 20;13:1026214. doi: 10.3389/fimmu.2022.1026214 (PMC9632486; doi:10.3389/fimmu.2022.1026214)

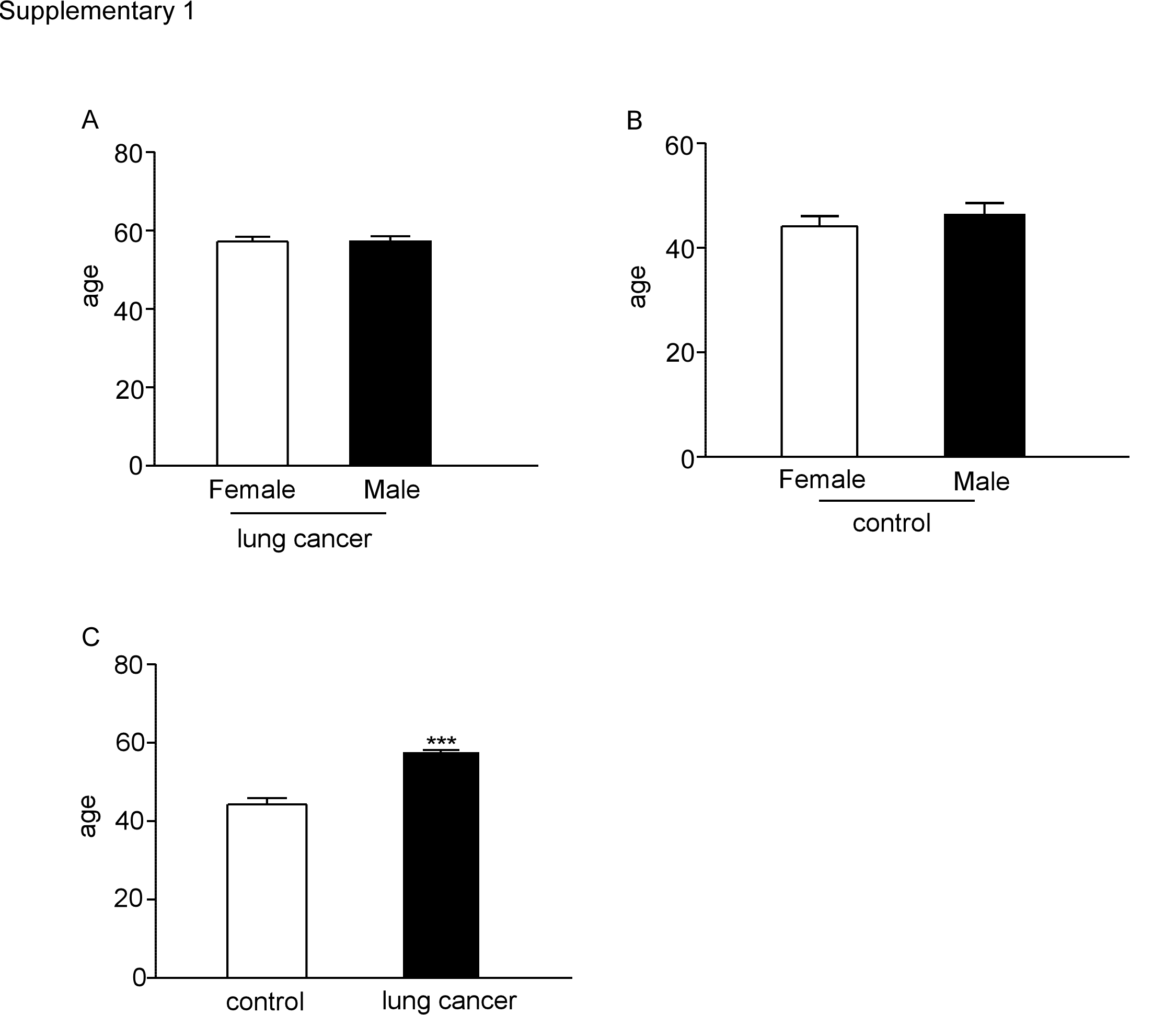

Supplement: Supplementary Figure 1 — Non-small cell lung cancer (NSCLC) and control sample information. (A) Analysis of sexes and age distributions from NSCLC. (B) The analysis of sexes and age distributions from control. (C) Analysis of age distributions for both NSCLC and control. ***P < 0.0001; Mann–Whitney test (two-tailed) and non-paired Student’s t-test. [file Image_1.tif]

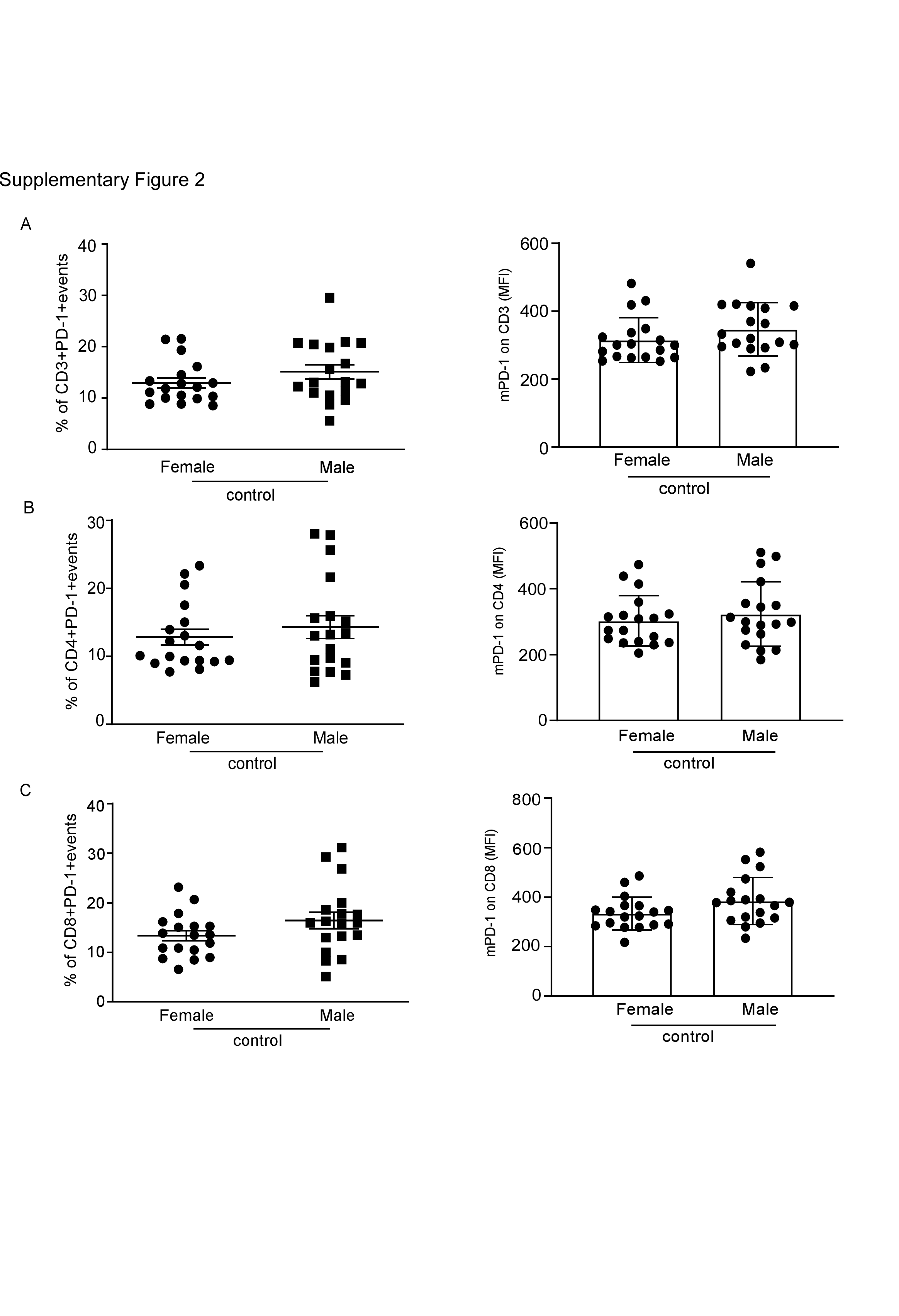

Supplement: Supplementary Figure 2 — The expression of mPD-1 on T cells in lung cancer patients and control at different ages. The percentage and MFI of CD3+PD-1+T cell (A), CD4+PD-1+T cell (B), and CD8+PD-1+T cell (C) in peripheral blood mononuclear cells (PBMCs) from control. The MFI of the CD3+PD-1+T cell (D), CD4+PD-1+T cell (E), and CD8+PD-1+T cell (F) in PBMCs from human lung cancer patients in young, middle-aged, and old subjects. The MFI of the CD3+PD-1+T cell (G), CD4+PD-1+T cell (H), and CD8+PD-1+T cell (I) in PBMCs from control in groups under 40 years old and above 40 years old. (J) Analysis of age distribution between men and women in the control. *P < 0.05, ***P < 0.001, ****P < 0.0001, Mann–Whitney test (two-tailed) and nonpaired Student’s t-test. Young, 21–40 years old age group; middle-aged, 41–64 years old age group; old subjects, 65+ years old; LC, lung cancer; MFI, mean fluorescence intensity. [file Image_2.tif]

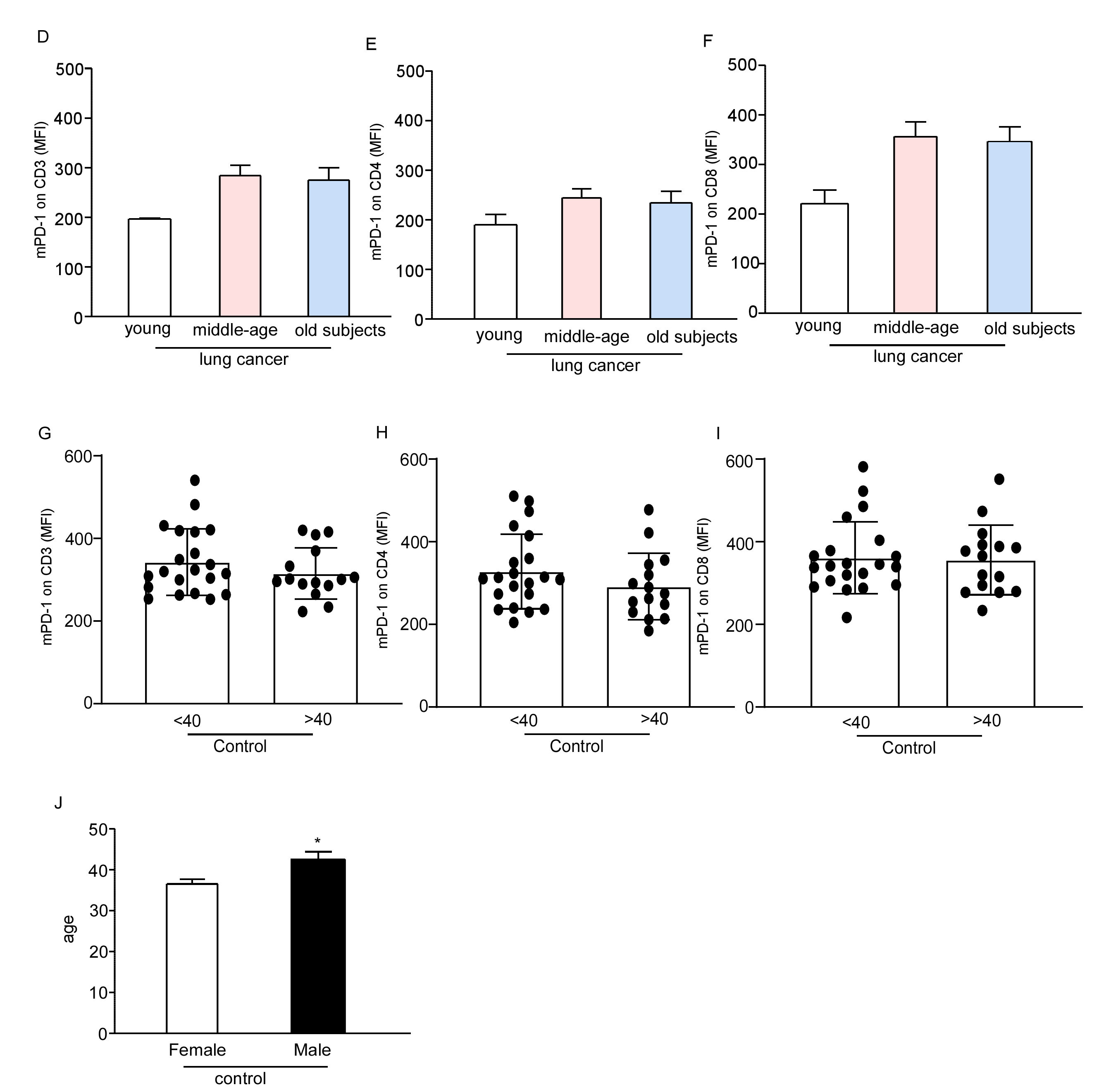

Supplement: Supplementary Figure 3 — Serum concentration of estradiol and testosterone in lung cancer patients. The serum estradiol and testosterone levels were examined by CMIA. (A) The serum concentration of estradiol (pg/ml) was analyzed in non-small cell lung cancer (NSCLC) patients. (B) The serum levels of estradiol (pg/ml) were detected in male NSCLC patients and control at different ages. (C) The serum concentration of testosterone (ng/ml) was analyzed in NSCLC patients. ***P < 0.0001; Mann–Whitney test (two-tailed) and non-paired Student’s t-test. E2, estradiol. [file Image_3.tif]

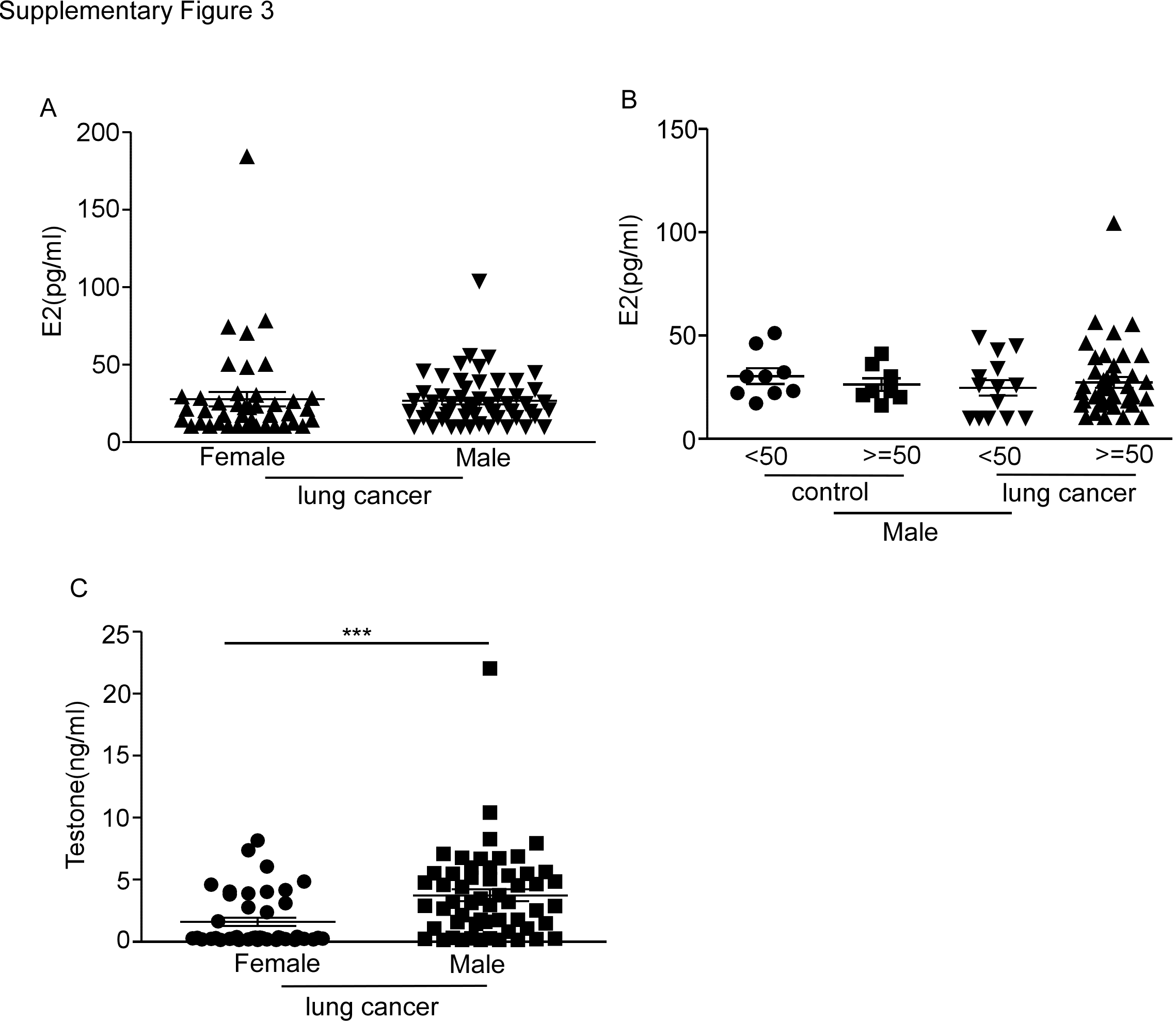

Supplement: Supplementary Figure 4 — The percentage of mPD-1+ CD4+/CD8+T cells from healthy control was analyzed by FACS. Peripheral blood mononuclear cells (PBMCs) were treated for 24 h with different concentrations of estrogen (10, 20, and 50 nmol) and androgen (1 and 10 nmol). (A) The frequency of CD8+PD-1+T cells (A) and CD4+PD-1+T cells (B) which were treated with the ratio of T/E at 1:50 (1 nmol androgen and 50 nmol estrogen), 1:20 (1 nmol androgen and 20 nmol estrogen), 1:10 (1 nmol androgen and 10 nmol estrogen), 1:5 (1 nmol androgen and 5 nmol estrogen), 1:2 (1 nmol androgen and 2 nmol estrogen), and 1:1 (1 nmol androgen and 1 nmol estrogen) (healthy donor, n = 3). *P < 0.05, Mann–Whitney test (two-tailed) and paired Student t-test. T/E, androgen/estrogen. [file Image_4.tif]

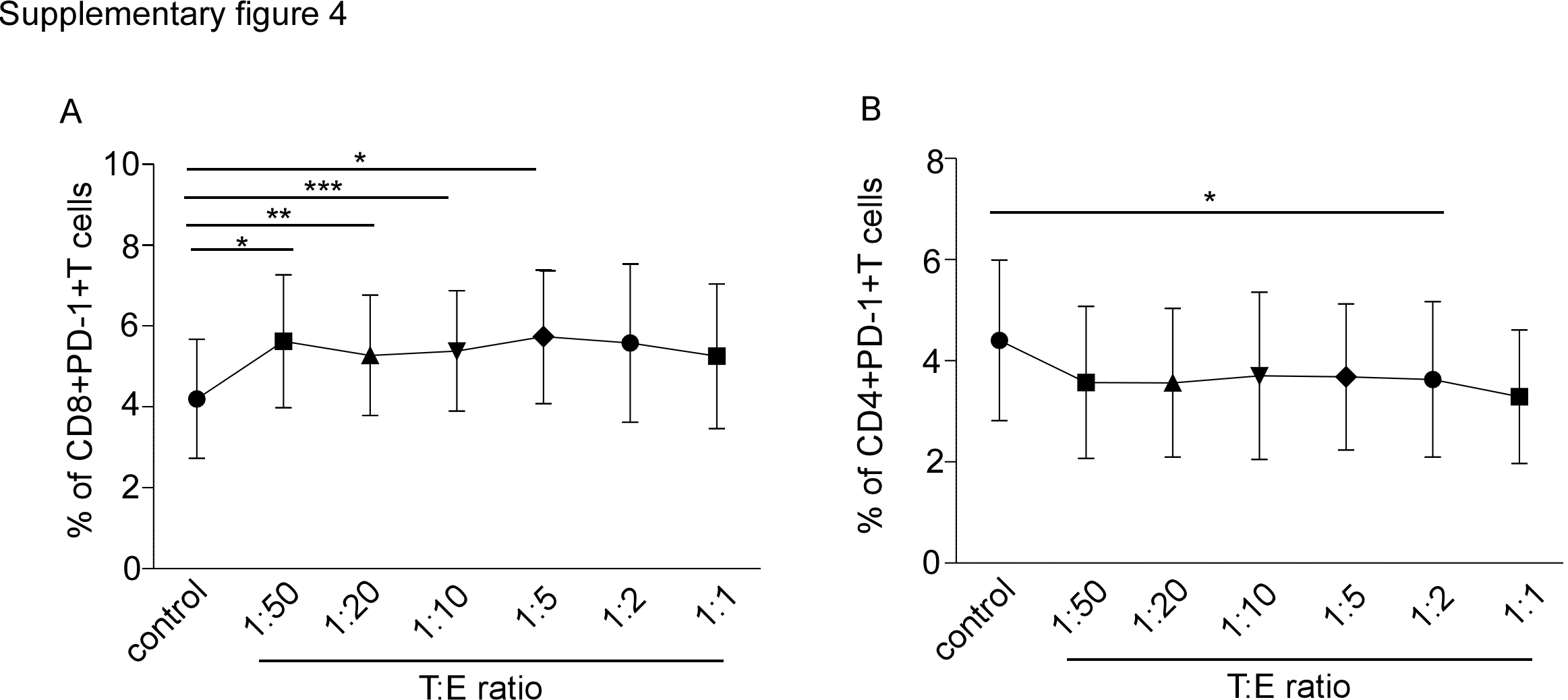

Supplement: Supplementary Figure 5 — Correlation analysis of the expression of mPD-1 and ER/AR on T cells. (A) Correlation between the expression of ER and mPD-1 on CD3 T cells, CD4 T cells, and CD8 T cells from non-small cell lung cancer (NSCLC) and control subjects. (B) Correlation between the expression of AR and mPD-1 on CD3 T cells, CD4 T cells, and CD8 T cells from NSCLC and control subjects. (C) Correlation between the expression of ER and AR on CD3 T cells, CD4 T cells, and CD8 T cells from NSCLC and control subjects. P-values were obtained by two-tailed Spearman’s test. Linear regression analysis was performed in GraphPad Prism to get the R 2 and P-values. R 2, goodness of fit; P, significance of the slope deviation from zero; NS, non-significant (n = 36). [file Image_5.tif]

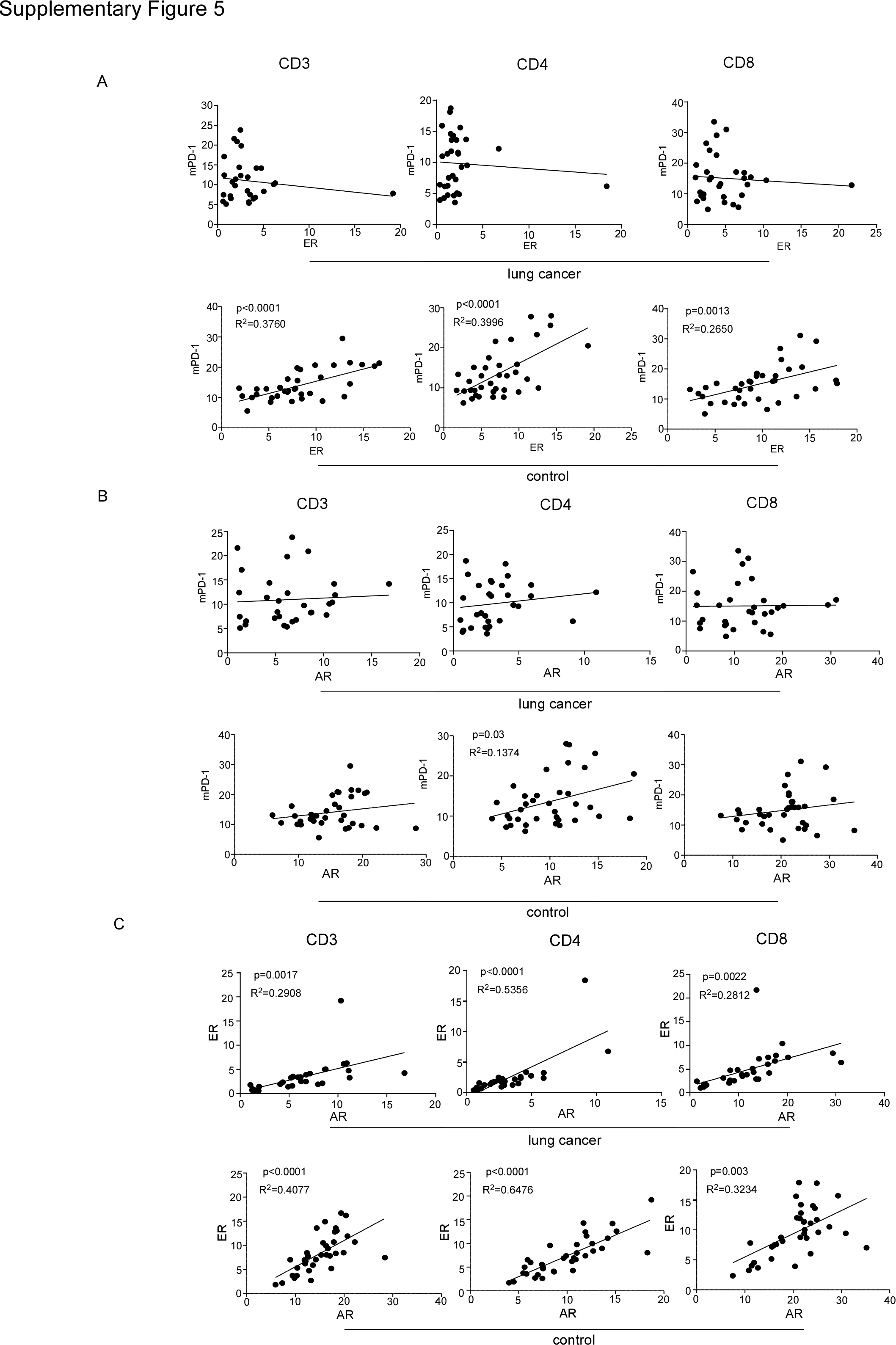

Supplement: Supplementary file 6 [file Image_6.tif]
